# Primary hemiarthroplasty for unstable osteoporotic intertrochanteric fractures in the elderly: A retrospective case series

**DOI:** 10.4103/0019-5413.67122

**Published:** 2010

**Authors:** KH Sancheti, PK Sancheti, AK Shyam, S Patil, Q Dhariwal, R Joshi

**Affiliations:** Sancheti Institute of Orthopaedics and Rehabilitation, Pune, Maharashatra, India

**Keywords:** Hemiarthroplasty, osteoporotic fractures, unstable intertrochanteric fractures

## Abstract

**Background:**

The management of unstable osteoporotic intertrochantric fractures in elderly is challenging because of difficult anatomical reduction, poor bone quality, and sometimes a need to protect the fracture from stresses of weight bearing. Internal fixation in these cases usually involves prolonged bed rest or limited ambulation, to prevent implant failure secondary to osteoporosis. This might result in higher chances of complications like pulmonary embolism, deep vein thrombosis, pneumonia, and decubitus ulcer. The purpose of this study is to analyze the role of primary hemiarthroplasty in cases of unstable osteoporotic intertrochanteric femur fractures.

**Materials and Methods:**

We retrospectively analyzed 37 cases of primary hemiarthroplasty performed for osteoporotic unstable intertrochanteric fractures (AO/OTA type 31-A2.2 and 31-A2.3 and Evans type III or IV fractures). There were 27 females and 10 males with a mean age of 77.1 years (range, 62–89 years).

**Results:**

Two patients died due to unrelated cause (myocardial infarction) within 6 months of surgery and remaining 35 patients were followed up to an average of 24.5 months (range,18–39 months). The average surgery time was 71 min (range, 55–88 min) with an average intraoperative blood loss of 350 ml (range, 175–500 ml). Six patients needed blood transfusion postoperatively. The patients walked on an average 3.2 days after surgery (range, 2–8 days). One patient had superficial skin infection and one had bed sore with no other significant postoperative complications. One patient of Alzheimer’s disease refused to walk and had a poor result. A total of 32 out of 35 patients (91%) had excellent to fair functional results and 2 had poor result with respect to the Harris hip score (mean 84.8±9.72, range 58-97). One patient who had neurological comorbidity refused to walk post operatively and was labeled as failed result.

**Conclusion:**

Hemiarthroplasty for unstable osteoporotic intertrochanteric fractures in elderly results in early ambulation and good functional results although further prospective randomized trials are required before reaching to conclusion.

## INTRODUCTION

There were an estimated 1.66 million hip fractures worldwide in 1990.^1^ This worldwide annual number is rising rapidly^2^,^3^ with an expected incidence of 6.26 million by the year 2050.^1^,^4^ An increase in these fractures is on the rise due to the increased life expectancy of the people and osteoporosis.^1^–^5^ The mechanism of injury is mostly trivial trauma. Bergström *et al*.^6^ found that low-energy trauma (fall<1 m) caused 53% of all fractures in persons 50 years of age and older. In those over 75 years, low-energy trauma caused>80% of all fractures. The contribution of osteoporosis-related fractures is more important than previously thought.

Stable fractures can be easily treated with osteosynthesis with predictable results. However, the management of unstable intertrochantric (Evans type III or IV and AO/OTA type 31-A2.2 and 2.3)^7^–^8^ fractures is a challenge because of difficulty in obtaining anatomical reduction.

In the past, fixed nail plate devices used for the fixation of these fractures had high rates of cut-out and fracture displacement.^9^–^11^ Subsequently, a sliding hip screw was used with much success and became the predominant method of fixation of these fractures.^12^–^15^ Complications such as head perforations, excessive sliding leading to shortening, plate pullout, and plate breakage continued to be a problem especially with the unstable type of fractures.^16^–^19^ Osteoporosis and instability are one of the most important factors leading to unsatisfactory results.^20^–^22^ Also in these elderly patients with unstable osteoporotic fractures, a period of restricted mobilisation is suggested,^23^,^24^ which may cause complications like atelectasis, bed sores, pneumonia, and deep vein thrombosis.^25^ Thus fracture stability, bone strength, and early rehabilitation determined the final results in cases of intertrochantric fractures.

Intramedullary interlocking devices have shown reduced tendency for cut-outs in osteoporotic bones 26,27 and also have better results in cases of unstable intertrochanteric fractures.^28^–^32^ However, the role of the intramedullary devices in unstable osteoporotic and severely comminuted intertrochanteric fractures is still to be defined. Endoprosthetic replacements have also been shown to achieve early rehabilitation of the patient and good long-term results.^33^–^37^ However, an ideal treatment method is still rather controversial because of the poor quality of bone mass, comorbid disorders, and difficulty in rehabilitation of these patients.^38^

The purpose of this study is to analyze the role of primary hemiarthroplasty in cases of unstable osteoporotic intertrochanteric femur fractures.

## MATERIALS AND METHODS

37 cases of intertrochantric fractures treated with hemiarthroplasty between March 2006 and December 2007 were studied retrospectivly. There were 27 females and 10 males. All patients were above the age of 60 years (range 62-89 years). All patients had confirmed osteoporosis on the preoperative bone mineral density scan confirming with the WHO criteria.^39^ The fractures were classified according to AO/OTA and Evans classification. Only AO/OTA type 31-A2.2 and 31-A2.3 and Evans type III or IV fractures were included in this study. Although the AO/OTA classification classifies these fractures as pertrochanteric, however since we also used the Evans classification we retained the terminology of the intertrochanteric fracture to avoid confusion. Patients with associated fractures that might significantly affect the final functional outcome, patients that were nonambulatory before injury and patients with psychiatric disorders were excluded from the study (*n*=1). All patients were community ambulators prior to trauma. Twenty were walking independently without support while rest of them required an aid like a cane or a walking stick. None of our patients had any significant preexisting hip pathology.

**Figure 1:**
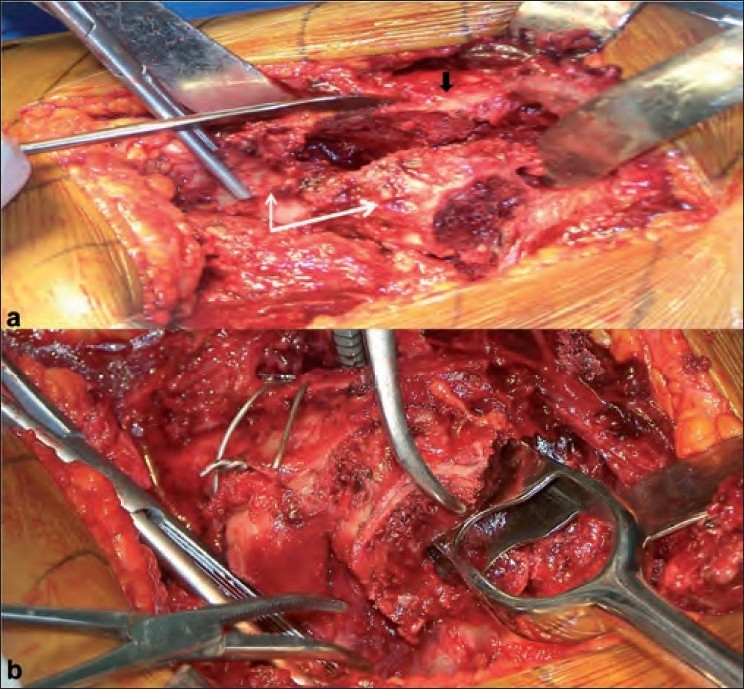
(a) Intraoperative photographs showing the lesser trochanter attached with the neck (white arrows) and the shaft fragment (black arrow) (b) The lesser trochanter and the shaft are tied together with steel wires to reconstruct the proximal femur and broaching is done

**Figure 2:**
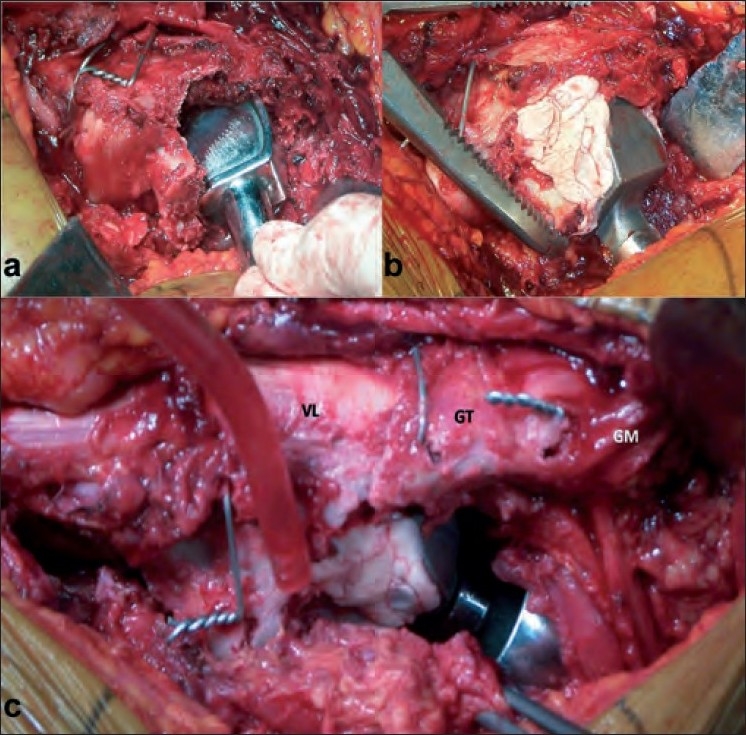
Intraoperative photographs showing (a) a stem placed in the broached canal to have an idea about the bone deficiency. (b) The deficient area is built up by the cement mantle. (c) The sleeve of the gluteus medius (GM), greater trochanter (GT), and vastus medialis (VM) is fixed to the proximal shaft with an additional tension band wire

**Figure 3:**
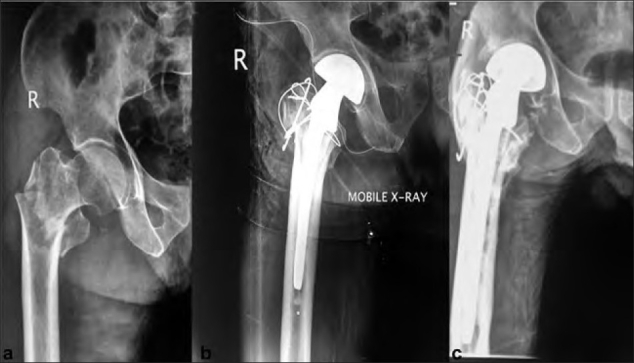
X-ray right hip joint anteroposterior view (a) 80-year-old male showing an unstable intertrochanteric fracture, (b) treated with primary partial hemiarthroplasty, (immediate postoperative) (c) 2-year follow-up radiographs

All cases were operated by using a standard posterior approach in lateral position by the senior author (KHS). The fracture anatomy was assessed and a cut was taken high up in the neck (almost subcapital level) to facilitate removal of the femoral head. With the removal of the head, the fracture now had three main fragments namely the greater trochanter, the lesser trochanter, and the shaft [Figure 1a] with the retained portion of the neck of femur. In 21 cases, the lesser trochanter was in continuity with the neck of the femur and was reconstructed with the shaft and greater trochanter using steel wires [Figure 1b]. A neck cut was then taken roughly about 1–2 cm above the lesser trochanter depending upon the amount of comminution. At times, the lesser trochanter was found as a separate fragment with neck as a separate fragment; in these cases, it was difficult to reconstruct the calcar (*n*=11). In these cases, the lesser trochanter and the greater trochanter were fixed to the shaft using steel wires; however, most of the portion of the neck had to be sacrificed. In five cases where the lesser trochanter was comminuted, the trochanter pieces were left attached to the soft tissue and the medial defect was reconstructed using a cement mantle (*n*=3). In 19 cases, the greater trochanter was the fracture en masse and was reattached to the main shaft using steel wires. In 9 cases where the greater trochanter was coronally split a tension band was applied beneath the gluteus medius tendon and a bony tunnel was drilled in the distal greater trochanter. In 7 cases, the greater trochanter was found to be severely comminuted; here ethibond sutures were used to suture together the trochanter pieces and the soft tissue to make a stable construct. The gluteus medius, greater trochanter, and the vastus lateralis apparatus were maintained in continuity as a stable lateral sleeve. This was then fixed loosely to the shaft fragment with steel wires or ethibond sutures. In cases where both greater and lesser trochanters were comminuted (*n*=2), they were both segregated together with the ethibond sutures to form separate masses and were reattached to the shaft after the insertion of a cemented femoral stem. Thus at the end of reconstruction, the greater trochanter, the lesser trochanter, and the shaft were wired together using steel wires in 32 cases while only ethibond sutures were used in five cases which were severely comminuted. The femoral canal was broached with appropriate anteversion [Figure 1b]. A fixed bipolar prosthesis was then inserted and trial reduction was done. With the trial prosthesis insitu traction was applied to the leg and compared with the opposite leg for limb length equality. After confirming the leg length the implant was inserted into the femur and joint was reduced. Traction was then applied with implant in situ to achieve the desired limb length by comparing with the opposite limb on table. Applied traction causes the femur to be pulled distally and, a note of distraction between the prosthesis and the femoral cut was made and the level on the prosthesis was marked. This gives an idea of how much the femur implant should sink into the proximal femur so as to achieve limb length at the time of final cementing of the implant. We used the second-generation cementing technique and cement restrictor in all cases. During the final fixation of the stem, the cemented stem was allowed to sink in the femoral canal up to the mark made on the prosthesis in previous step and for the remaining portion a cement mantle was made so that the final limb length was equalized [Figure 2]. This procedure was especially required in cases where the lesser trochanter was fractured separate from the neck portion. Cement was used for distal fixation also as the medullary canal was invariably found to be very wide. Once the prosthesis was fixed, the broken trochanter and calcar were again retightened by tensioning the wire cables. The sleeve of gluteus medius, greater trochanter, and vastus medialis if reconstructed was now reattached to the shaft by additional wires [Figure 3]. The short external rotators were then sutured back using bone tunnels in the greater trochanter with the closure of the superficial layers, as routine over a suction drain after achieving hemostasis.

All patients underwent a routine postoperative physiotherapy protocol that included early gait training in form of walking with the help of a walker starting second day post surgery. The rehabilitation then progressed as tolerated by the patients. Patients were examined postoperatively at 6 weeks, 3 months, 6 months, 1 year, and thereafter annually. At each follow-up visit, a clinicoradiological examination was done and the patient was evaluated using the Harris hip score (HHS) and were graded as <70 poor, 70-79 Fair, 80-89 Good and 90-100 Excellent. Anteroposterior radiographs of the hip were analyzed at each follow-up to note evidence of loosening.

## RESULTS

**Figure 4:**
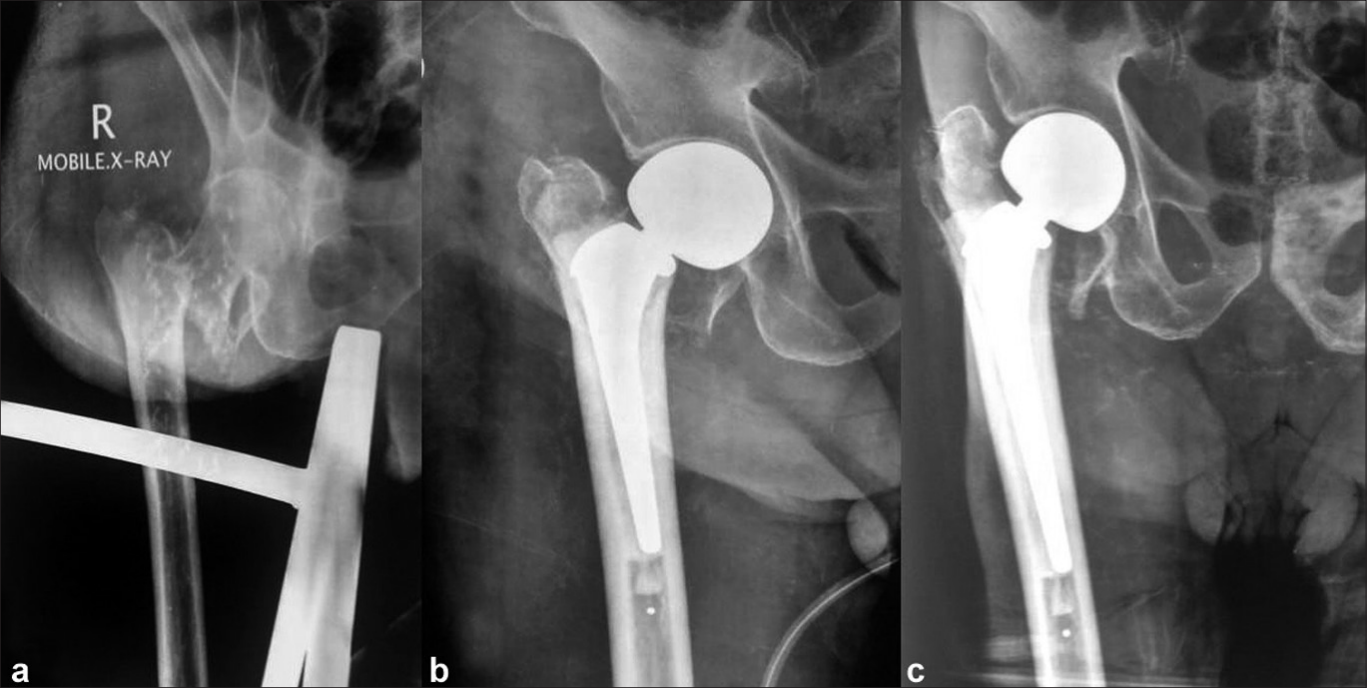
X-ray right hip joint anteroposterior view in (a) an 87 year old man with severe osteoporosis and unstable intertrochanteric fracture (b) treated with hemiarthroplasty (This patient had Alzheimer’s disease and refused to walk and eventually was wheelchair bound) (c) with adduction contrauture

The average age of patients was 77.1 years (range, 62–89 years). Nine of our patients had associated comorbidities (hypertension, *n*=5 and diabetes, *n*=4). Twenty of our patients were walking independently without support before the fracture. All patients were operated within 15 days (mean delay of 5.61±3.73 days, range 2 days to 14 days) with delay due to patients presenting late and time taken for patients to be fit for anaesthesia. The average surgery time was 71 min (range, 55–88 min) with an average intraoperative blood loss of 350 ml (range, 175–500 ml). Six patients needed single unit blood transfusion each postoperatively, rest of the patients did not require any blood transfusion. The patients started full weight bearing at an average 4.2 days after surgery (range, 3–8 days). One patient refused to walk after surgery and had a poor result (HHS 58). The average stay in the hospital was 10.96 days (range, 5–21 days). One of the patients developed bed sore postoperatively, and required a week more of hospital stay, till the healing of the sore. This patient was operated on 5 ^th^ day post injury and did not have a pre operative bed sore. Out of the 37, two patients expired due to unrelated causes (both due to myocardial infarction). The first among these patients was an 85 year old female with hypertension, diabetes and ischemic heart disease and was operated on 8 day post trauma. She died 3 months after surgery due to myocardial infarction. The second patient was 78 year old male with ischemic heart disease and right nephrectomy and chronic renal failure, was operated on day 4 post injury and died 5 months post surgery due to myocardial infarction. The remaining 35 patients having a minimum one year follow up were evaluated and data was further analyzed for only these 35 patients. The minimum follow up was average of 24.5 months (range, 18–39 months). One patient developed pneumonia which settled down with intravenous antibiotics. One patient had a periprosthetic fracture 6 months after surgery which was treated with a locking compression plate. The fracture healed and the patient went on to have an excellent result. At the end of 3 months, 7 patients were graded as excellent, 16 patients as good, 9 patients as fair, 2 patients as poor, and 1 patient as failed. At latest follow-up (mean 24.5 months, range 18 months to 39 months), the mean HHS was 84.8±9.72 (range, 58–97). A total of 10 patients were graded as excellent, 15 patients as good, 7 as fair, 2 as poor, and 1 as failed. Preinjury 20 patients were walking without support, 17 patients were walking with support (cane *n*=5, walker *n*=2). At last follow-up, 23 patients were walking without any aid, 10 patients had a limp and used a stick for walking, 1 patient used a walker, and 1 was wheelchair bound. Ten patients had shortening of the operated limb with an average shortening of 1.1 cm (range, 5–15 mm) which was well compensated by giving a shoe raise. A total of 22 patients had an abductor lurch at 3-month follow-up; however, only 6 patients had abductor muscle weakness with a positive Trendelenberg test at final follow-up. Most of these patients however could walk well with the use of a stick. One patient had Booker grade 1 heterotropic ossification 40 at 6-month follow up; however, this did not restrict the range of motion. Among the patients with poor results, one patient had a superficial wound infection which settled down with a course of intravenous antibiotics for 2 weeks. However, the patient continued to have diffuse pain along the incision site and walked with a limp. The second patient of poor results also had pain and limp, but we could not find any obvious reason for the pain. The patient with the failed result was a case of Alzheimer’s disease. The patient did not cooperate with the physiotherapy program and refused to walk postoperatively. Eventually, the patient developed a severe adduction contracture and was wheelchair bound [Figure 4]. There were no dislocation, loosening, or late infections.

## DISCUSSION

Hip fractures are associated with notable morbidity and mortality in elderly patients. Internal fixation has drastically reduced the mortality associated with intertrochantric fractures; 41 however, early mobilization is still avoided in cases with comminution, osteoporosis, or poor screw fixation.^42^,^43^ Primary hemiarthroplasty offers a modality of treatment that provides adequate fixation and early mobilization in these patients thus preventing postoperative complications such as pressure sores, pneumonia, atelectasis, and pseudo arthrosis.^44^,^25^ The Indian perspective regarding the use of primary arthroplasty as a modality of treatment for severe comminuted unstable intertrochantric fractures is been commented on by few authors;^45^,^46^ however, ours is the first case series reporting the Indian experience with this technique.

Hemiarthroplasty has been used for unstable intertrochanteric fractures since 1971,^25^ however less frequently as compared to femoral neck fractures.^47^ Its initial use was as a salvage procedure for failed pinning or other complications.^48^ Tronzo claimed to be the first to use long, straight-stemmed prosthesis for the primary treatment of intertrochanteric fractures.^33^ Rosenfeld, Schwartz, and Alter reported good results with the use of the Leinbach prosthesis.^49^ Since then there are multiple studies showing good results using this technique. Stern and Goldstein used the Leinbach prosthesis for the primary treatment of 22 intertrochantric fractures and found early ambulation and early return to the prefracture status as a definite advantage.^48^ Liang *et al*.^50^ in their study of unstable intertrochanteric fractures concluded hemiprosthesis arthroplasty is an effective method to treat the unstable intertrochanteric fractures in elderly. It can decrease the complications, reduce the mortality, improve the patient’s living quality, and reduce the burden of the patient’s family. Grimsrud *et al*.^51^ studied 39 consecutive patients of unstable intertrochanteric fractures treated with a cemented bipolar hip arthroplasty. They concluded that these fractures can be treated with a standard femoral stem and cerclage cabling of the trochanters. The technique allows safe and early weight bearing on the injured hip and had a relatively low rate of complications. In our series too there was only one case of pressure sores which healed with conservative management. Since most of the patients were out of bed on the second day postoperatively, and the recumbancy time was minimal, there were no chest complications in our series. Rodop *et al*.^52^ in a study of primary bipolar hemiprosthesis for unstable intertrochanteric fractures in 37 elderly patients obtained 17 excellent (45%) and 14 good (37%) results after 12 months according to the Harris hip-scoring system. A total of 25 out of 35 patients in our study had a good to excellent result (71%). If the patients with a fair result were also included, the percentage goes up to 91%. Thus the results of this modality of treatment are definitely promising especially in view of the variable results of osteosynthesis in this group.^20^ The opponents of the technique stated increase blood loss, mechanical complications like dislocation, and infection as possible complications as compared to conventional internal fixation. The earliest comparison of internal fixation and hemiarthroplasty was done by Haentjens *et al*.^53^ showing a significant reduction in the incidence of pneumonia and pressure sores in those undergoing prosthetic replacement. In a comparative study of cone hemiarthroplasty versus internal fixation, Kayali *et al*.^54^ reached the conclusion that clinical results of both groups were similar. Hemiarthroplasty patients were allowed full weight bearing significantly earlier than the internal fixation patients. Broos *et al*.^36^ concluded that the operative time, blood loss, and mortality rates were comparable between the two groups, with a slightly higher percentage (73% versus 63%) of those receiving a prosthesis considered to be pain free. The functional outcome was comparable between both groups. Stappaerts *et al*.^55^ found no difference between two groups except a higher transfusion need in the replacement group. In our series the average blood loss was 350 ml with only six patients requiring postoperative blood transfusion and there was no incidence of dislocation.

Conflicting reports about postoperative mortality in cases with primary hemiarthroplasty are cited in the literature. Kesmezacare *et al*.^56^ reported postoperative mortality in 34.2% after a mean of 13 months and in 48.8% after a mean of 6 months in patients treated with internal fixation and endoprosthesis, respectively. Other studies have shown no differences in postoperative mortality in two groups.^36^,^53^,^55^ In present series only 2 patients out of the 37 died (5.4%) within 6 months of surgery due to unrelated causes (both secondary to myocardial infarction).

Hardy *et al*.^57^ reported early weight bearing without excessive collapse in cases with comminuted intertrochantric fractures fixed with intramedullary nailing. However, there is only one study by Kim *et al*.^58^ which compares the calcar replacement prosthesis with intramedullary nailing in a prospective study involving two groups of 29 patients. They could not find any significant difference concerning the functional outcomes, but the cut-out rate of the hip screw was 7% in their patients. The Cochrane database analysis of relevant studies concluded that there is insufficient evidence to prove that primary arthroplasty has any advantage over internal fixation.^47^ However, they also mentioned that there were only two randomized trials studied and both had methodological limitations, including an inadequate assessment of the longer term outcome.

Delay in surgery is an important predictor for mortality in patients with proximal femur fracture and also of the postoperative morbidity^59^,^60^. We in our study, however, could not comment on these points because of small sample size and this is one of the limitations of our study. Further, inhomogeneous population in terms of existing co-morbidity and retrospective nature of our study are the other limitations.

Thus in conclusion, primary hemiarthroplasty does provide a stable, pain-free, and mobile joint with acceptable complication rate as seen in our study; however a larger prospective randomised study comparing the use of intramedullary devices against primary hemiarthroplasty for unstable osteoporotic fractures will be needed.
